# Combination of ACY-241 and JQ1 Synergistically Suppresses Metastasis of HNSCC via Regulation of MMP-2 and MMP-9

**DOI:** 10.3390/ijms21186873

**Published:** 2020-09-19

**Authors:** Ha Young Cho, Sang Wu Lee, Yu Hyun Jeon, Dong Hoon Lee, Go Woon Kim, Jung Yoo, So Yeon Kim, So Hee Kwon

**Affiliations:** 1 College of Pharmacy, Yonsei Institute of Pharmaceutical Sciences, Yonsei University, Incheon 21983, Korea; hayoung.cho@yonsei.ac.kr (H.Y.C.); tkddn407@naver.com (S.W.L.); hyun953@naver.com (Y.H.J.); tci30@naver.com (D.H.L.); goun6997@daum.net (G.W.K.); jungy619@yonsei.ac.kr (J.Y.); ksy_dct@naver.com (S.Y.K.); 2 Department of Integrated OMICS for Biomedical Science, Yonsei University, Seoul 03722, Korea

**Keywords:** HNSCC, HDAC6, ACY-241, JQ1, metastasis, HPV

## Abstract

Overexpression of histone deacetylase 6 (HDAC6) and bromodomain-containing protein 4 (BRD4) is related to aggressiveness of head and neck squamous carcinoma (HNSCC). Based on studies that HDAC6 and BRD4 are potential therapeutic targets of HNSCC, we hypothesized that the combination treatment of BET inhibitor JQ1 and HDAC6-selective inhibitor ACY-241 could exhibit synergistic anticancer effects in human papillomavirus (HPV)-positive and HPV-negative HNSCC cells. In this study, HNSCC cell growth and viability were measured by CCK-8 assay, apoptosis was analyzed by flow cytometry, and metastasis was studied by wound healing and transwell assays. Furthermore, immunoblotting is conducted to investigate proteins that modulate apoptosis or metastasis. Here, we report that the combination of ACY-241 and JQ1 shows synergistic cell growth inhibition, viability reduction, and apoptosis induction in HNSCC cells through inactivation of AKT and NF-κB signaling. Importantly, we demonstrate that combined treatment of ACY-241 and JQ1 synergistically suppresses TNF-α-induced migration and invasion via dysregulating matrix metalloproteinase (MMP)-2, MMP-9, and MT1-MMP. Overall, the combination of ACY-241 and JQ1 significantly suppresses proliferation and metastasis in HPV-positive and HPV-negative HNSCC. Collectively, these findings suggest that the co-inhibition of BET and HDAC6 can be a new therapeutic strategy in HNSCC.

## 1. Introduction

Head and neck cancer is the sixth most common cancer worldwide and occurs in the head and neck area, including the larynx, nasal cavity, oral cavity, paranasal sinuses, pharynx, and salivary glands. Despite various primary tumor sites, more than 95% of these epithelial tumors are head and neck squamous cell carcinoma (HNSCC) [1]. Although human papillomavirus (HPV) infection is not the major risk factor of all HNSCCs [2,3], HPV-positivity relates to improved response to treatments and survival rates in a subset of HNSCCs [4]. In particular, HPV-16 is strongly associated with the development of oropharyngeal squamous cell carcinoma (OPSCC), and 71% of node metastatic OPSCC are detected with HPV-16 [5]. Interestingly, HPV-positive HNSCCs are more metastatic to cervical lymph nodes compared to HPV-negative tumors [6]. Distant metastasis is rare in HNSCC compared to other cancers such as stomach, pancreas, lung, breast, or kidney [7], but increasing incidence of HPV-positive HNSCC and notably low 5-year overall survival of patients with multiple metastases suggest the need to develop metastasis-directed therapy [8].

Many efforts have been put into finding the molecular mechanism of HNSCC metastasis. Metastatic tumor cells undergo activation of epithelial–mesenchymal transition (EMT), which includes the reorganization of extracellular matrix (ECM). Proteolysis of ECM is an essential process for malignant tumor metastasis and takes place by a group of zinc-dependent enzymes called the matrix metalloproteinase (MMP) family [9]. MMPs have been therapeutic targets for metastatic tumors by specific MMP inhibitors, as well as epigenetic agents such as histone deacetylase (HDAC) and bromodomain and extraterminal (BET) inhibitors [10,11,12]. 

HDACs are involved in several stages of cancer progression, and their overexpression leads to advanced stages and poor outcomes in patients. A high level of HDAC6 has been found in multiple cancers, including pancreatic cancer and leukemia [13,14]. HDACs are attractive targets of cancer therapy because they can reversely regulate acetylation of α-tubulin and stability of microtubules, increasing cell motility and cell cycle progression to accelerate proliferation, metastasis, and invasion [15]. HDAC inhibitors have been FDA-approved and are under clinical trials for various cancer treatments. Furthermore, HDAC6-selective inhibitors have been designed to overcome the toxicity of pan-HDAC inhibitors in cancer clinical trials. ACY-241, the second generation of HDAC6-selective inhibitor, has shown anti-tumor effects as a single agent and in combination with other anticancer agents [16]. High protein and mRNA levels of HDAC6 are detected in oral squamous cell carcinoma (OSCC), and inhibition of HDAC6 synergistically induces autophagy and apoptosis together with Bortezomib in HNSCC [17,18].

Human bromodomain (BRD) proteins of the BET family (BRD2, BRD3, BRD4, BRDT) recognize acetylated histone codes and modulate transcriptional elongation [19]. A high level of BRD4 is found in super-enhancers of tumor cells, which are 15-fold larger and notably more active than typical enhancers. BET inhibitors such as JQ1 decrease BRD4 binding at super-enhancers, thereby disturbing transcription of MYC oncogene and following MYC-dependent target genes [20]. Anti-tumor effects of JQ1 have been reported in solid cancers [21,22]. In HNSCC, overexpression of BRD4 is relevant to tumor aggressiveness and progression. As a result, JQ1 impairs cell proliferation and metastasis while inducing apoptosis and cell cycle arrest in HNSCC [12]. Since monotherapy of BET inhibitors has moderate effects with undeniable resistance and toxicity, efforts have been put into enhancing its potential as a therapeutic agent by combinatorial approach [23]. 

In this study, we asked whether co-targeting HDAC6 and BET by specific small molecule inhibitors would synergistically induce anticancer effects in HNSCC. We focused on HDAC6-selective inhibitor ACY-241 and BET inhibitor JQ1 to investigate anti-metastasis in HPV-positive and HPV-negative HNSCC cells. Our work demonstrates that the combination treatment of ACY-241 and JQ1 to HNSCC cells increases apoptosis while suppressing cell growth, viability, migration, and invasion. We also aim to investigate whether both HPV-positive and HPV-negative HNSCC cells respond to the epigenetic inhibitors. Our findings identify a novel therapeutic strategy for HNSCC using a combination of HDAC6 and BET inhibitors. Together, we suggest a potential combination therapy for HNSCC that can exhibit synergistic therapeutic effects while overcoming the limitations of single inhibitor treatments.

## 2. Results

### 2.1. ACY-241 and JQ1 Treatments Suppress Cell Growth and Synergistically Reduce Cell Viability in HNSCC Cells

**Figure 1:**
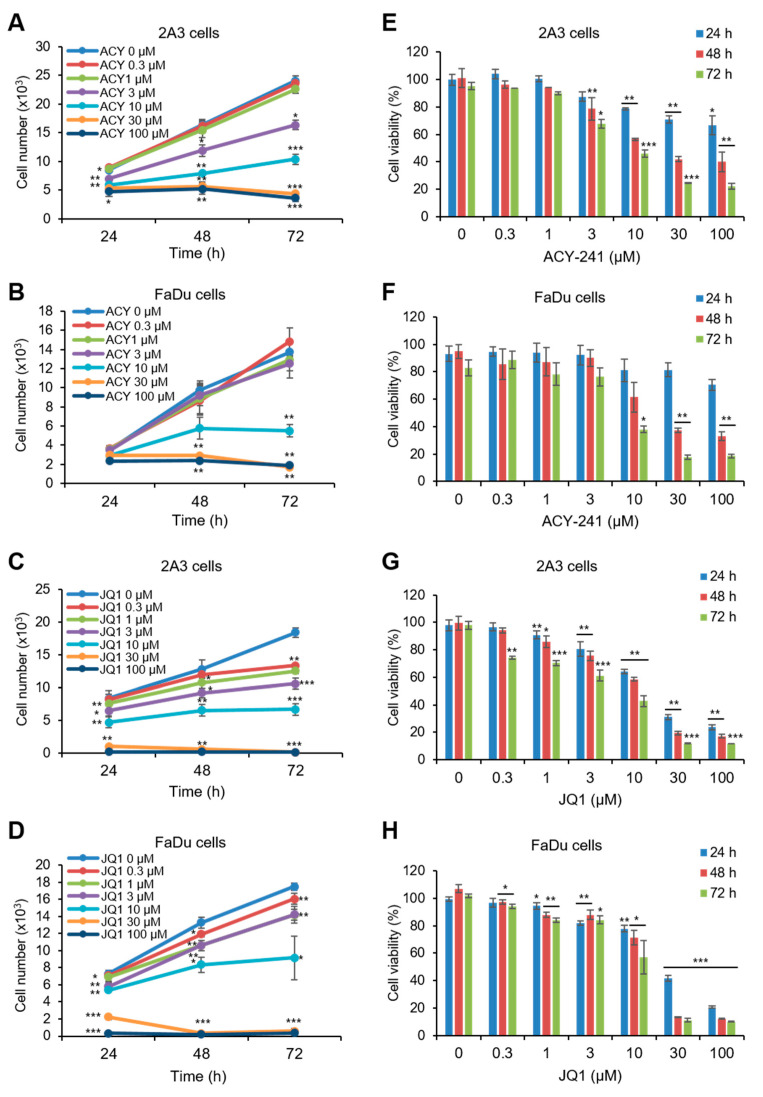
Single treatments of ACY-241 and JQ1 time- and dose-dependently suppress cell growth and reduce cell viability in both human papillomavirus (HPV)-positive and HPV-negative head and neck squamous carcinoma (HNSCC) cells. (**A**,**E**) 2A3 cells and (**B**,**F**) FaDu cells were treated with 0.1% DMSO or ACY-241 at indicated concentrations for 24–72 h. (**C**,**G**) 2A3 cells and (**D**,**H**) FaDu cells were treated with 0.1% DMSO or JQ1 at indicated concentrations for 24–72 h. CCK-8 assay was performed to measure cell growth (**A**–**D**) and viability (**E**–**H**). Values represent mean ± SD from three independent experiments (*n* = 3). * *p* < 0.05, ** *p* < 0.01, or *** *p* < 0.001 vs. DMSO control.

We conducted CCK-8 assay to investigate changes in cell growth and viability. First, HPV-positive 2A3 and HPV-negative FaDu HNSCC cells were treated with HDAC6-selective inhibitor ACY-241 and BET inhibitor JQ1 as monotherapy. Both 2A3 and FaDu cells showed a time- and dose-dependent decrease in cell growth and viability (Figure 1A–H). Half maximal growth inhibition concentration (GI_50_) and half maximal inhibitory concentration (IC_50_) were smaller in 2A3 cells than in FaDu cells after 72 h of inhibitor treatments (Table 1). This result shows that ACY-241 and JQ1 exhibit anti-proliferative effects in HNSCC cells regardless of HPV infection.

**Figure 2:**
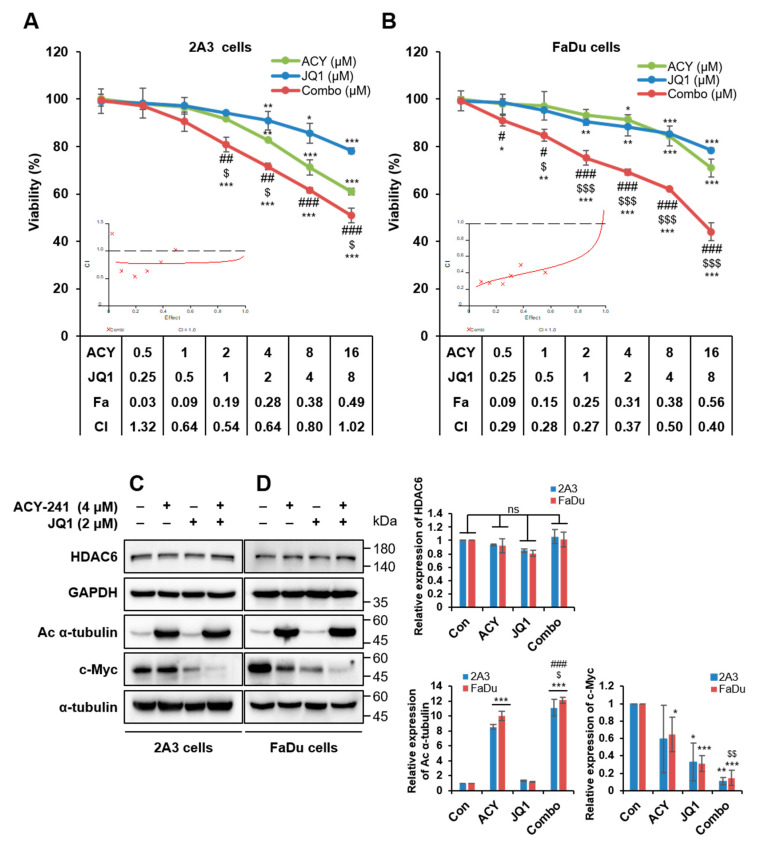
Combination treatment of ACY-241 and JQ1 synergistically decreases HNSCC cell viability. (**A**,**B**) ACY-241 and JQ1 were treated alone or in combination at a 2:1 ratio to 2A3 cells and FaDu cells. Cell viability was measured by CCK-8 assay (48 h). Synergism of ACY-241 and JQ1 was determined by combination index (CI) using Chou–Talalay method. (**C**,**D**) Inhibitory enzymatic effects of ACY-241 and JQ1 were confirmed by immunoblot analysis of acetyl α-tubulin and c-Myc, respectively. α-tubulin and GAPDH were used as loading controls. Protein levels were quantified relative to the loading control. Total protein was extracted after 24 h of ACY-241 (4 μM) or JQ1 (2 μM) treatment alone or in combination. Values represent mean ± SD (*n* = 3). * *p* < 0.05, ** *p* < 0.01, or *** *p* < 0.001 vs. DMSO control, ^$^ *p* < 0.05, ^$$^ *p* < 0.01, or ^$$$^ *p* < 0.001 vs. ACY-241-treated group, ^#^ *p* < 0.05, ^##^ *p* < 0.01, or ^###^ *p* < 0.001 vs. JQ1-treated group. ns = not significant.

Next, we treated ACY-241 and JQ1 in combination to HPV-positive and HPV-negative HNSCC cells and analyzed the cell viability using CCK-8 assays. A substantial decrease in viability was observed following combined treatment compared with single agents. Synergism was evaluated using the Chou and Talalay method [24]. The combination of ACY-241 and JQ1 showed synergistic cytotoxicity with a combination index (CI) of <1.0 (Figure 2A,B). These data confirmed the robust anti-proliferative effect when ACY-241 and JQ1 were combined in both HPV-positive and HPV-negative HNSCC cells. 

### 2.2. Combination Treatment of ACY-241 and JQ1 Synergistically Induces Apoptosis in HNSCC Cells

**Figure 3:**
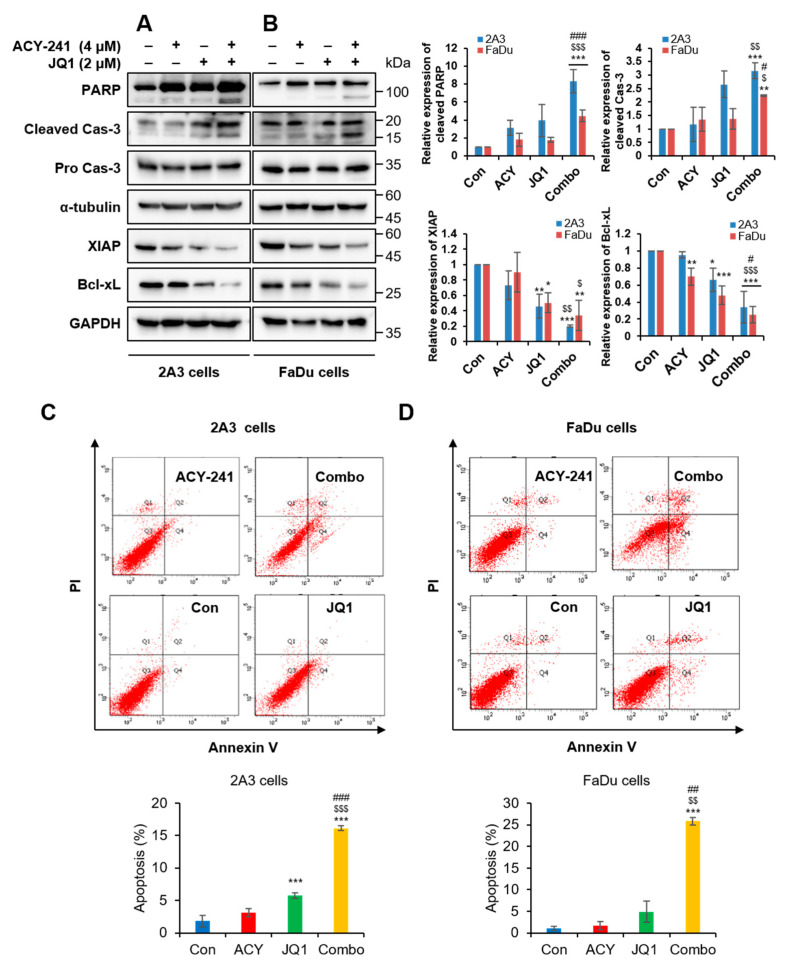
Combination treatment of ACY-241 and JQ1 synergistically induces apoptosis in HNSCC. (**A**,**B**) Immunoblot analysis of pro-apoptotic proteins (PARP, Cas-3) and anti-apoptotic proteins (XIAP, Bcl-xL) in 2A3 and FaDu cells. α-tubulin and GAPDH were used as loading controls. Protein levels were quantified relative to the loading control. Total protein was extracted after 24 h of ACY-241 (4 μM) or JQ1 (2 μM) treatment alone or in combination. (**C**,**D**) Flow cytometry analysis of 2A3 and FaDu cells. Cells were treated with 0.2% DMSO, ACY-241 (4 μM), or JQ1 (2 μM) alone or in combination for 72 h. 2A3 and FaDu cells were stained with annexin V and PI for 15 min. Values represent mean ± SD (*n* = 3). * *p* < 0.05, ** *p* < 0.01, or *** *p* < 0.001 vs. DMSO control, ^$^ *p* < 0.05, ^$$^ *p* < 0.01, or ^$$$^ *p* < 0.001 vs. ACY-241-treated group, ^#^ *p* < 0.05, ^##^ *p* < 0.01, or ^###^ *p* < 0.001 vs. JQ1-treated group.

Based on our results, further experiments were conducted with 4 µM of ACY-241 and 2 µM of JQ1, which is the combination of the lowest concentrations that display noticeable synergistic effect. First, enzymatic inhibitory activities of ACY-241 and JQ1 were confirmed by observing their target proteins, acetyl α-tubulin and c-Myc, respectively [25,26]. ACY-241 increased acetylation of α-tubulin, and JQ1 decreased c-Myc in both HPV-positive 2A3 and HPV-negative FaDu HNSCC cells. Furthermore, HDAC6 protein level remained unchanged by ACY-241 (Figure 2C,D). It has been previously reported that JQ1 did not modify BRD4 protein level [27]. We also confirmed that mRNA levels of HDAC6, BRD2, and BRD4 were unaffected after ACY-241 and JQ1 treatments (Figure S1A–C). As c-Myc oncogene is known to induce proliferation [20], we next performed immunoblotting to determine whether ACY-241 and JQ1 disrupt the apoptotic signaling pathway. PARP and caspase-3 were synergistically cleaved by combination treatment to exhibit pro-apoptotic effects. On the other hand, expression levels of anti-apoptotic proteins XIAP and Bcl-xL were synergistically reduced in both HPV-positive and HPV-negative HNSCC cells (Figure 3A,B). However, Bcl-2 associated pro-apoptotic proteins, such as Bak, Bax, and Bad, remained unchanged by ACY-241 and JQ1 combination (Figure S2). To further determine the apoptotic effect of HDAC6 and BET inhibition, flow cytometry analysis was performed to examine apoptosis after annexin V/propidium iodide staining. After 72 hours of combination treatment, early and late apoptosis were synergistically promoted in both HPV-positive and HPV-negative HNSCC cells. The percentage of apoptotic cells was as much as 9-fold higher than the additive effect of single inhibitor treatments (Figure 3C,D). Collectively, these data show that simultaneous inhibition of HDAC6 and BET is an effective treatment strategy to promote apoptosis in both HPV-positive and HPV-negative HNSCC cells. 

### 2.3. Combination Treatment of ACY-241 and JQ1 Synergistically Inhibits TNF-α-Induced Effects by Degrading MMP-2 and MMP-9

**Figure 4:**
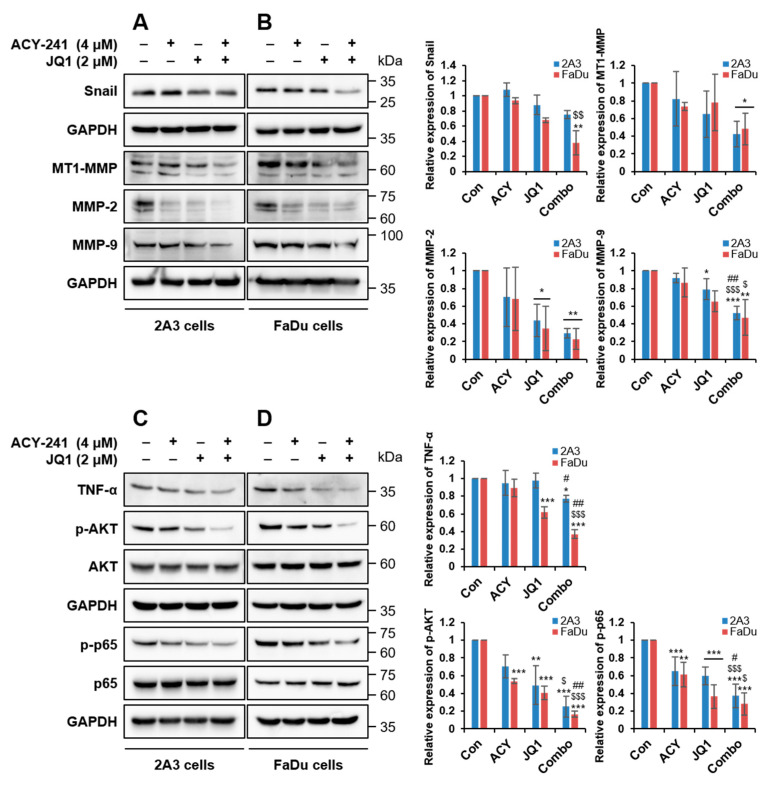
Combination treatment of ACY-241 and JQ1 synergistically downregulates MMP-2 and MMP-9 via inhibition of TNF-α/AKT/-NF-κB cascade in HNSCC. (**A**,**B**) Immunoblot analysis of Snail and MMP proteins (MMP-2, MMP-9, MT1-MMP) in 2A3 and FaDu cells. (**C**,**D**) Immunoblot analysis of EMT signaling factors (TNF-α, AKT, NF-κB) in 2A3 and FaDu cells. GAPDH was used as a loading control. Protein levels were quantified relative to the loading control. Total protein was extracted after 24 h of ACY-241 (4 μM) or JQ1 (2 μM) treatment alone or in combination. Values represent mean ± SD (*n* = 3). * *p* < 0.05, ** *p* < 0.01, or *** *p* < 0.001 vs. DMSO control, ^$^ *p* < 0.05, ^$$^ *p* < 0.01, or ^$$$^ *p* < 0.001 vs. ACY-241-treated group, ^#^ *p* < 0.05 or ^##^ *p* < 0.01 vs. JQ1-treated group.

To investigate the effect of HDAC6 and BET inhibition in metastasis, we tested protein expressions of the MMP family by immunoblotting. The most significantly associated MMPs in metastatic HNSCC are membrane-type 1-matrix metalloproteinase (MT1-MMP), MMP-1, -2, -3, and -9 and tissue inhibitors of metalloproteinase-2 [9,10]. EMT is a crucial cellular program that is observed during cell migration and wound healing, and it is regulated by MMP proteins [28]. Single treatments of ACY-241 and JQ1 substantially downregulated MMP-2, rather than MMP-9, in both HPV-positive 2A3 and HPV-negative FaDu HNSCC cells (Figure 4A,B). The combination of ACY-241 and JQ1 also synergistically reduced MMP-2 and MMP-9, the type IV collagenase that has a significant function in EMT by altering cell–matrix and cell–cell interactions [29]. Furthermore, this combination suppressed MT1-MMP, the activator of MMP-2 that is correlated with tumorigenesis of HNSCC [10] (Figure 4A,B). Consistent with protein levels, mRNA levels of MT1-MMP and MMP-2 were synergistically reduced by the combination treatment, but not MMP-9 (Figure S1D–F). In addition to MT1-MMP, tumor necrosis factor-α (TNF-α), an inducer of MMP-9 secretion in hypopharyngeal and OSCC cell lines [30], was synergistically suppressed by a combination of ACY-241 and JQ1 in both HNSCC cells (Figure 4C,D and Figure S1G).

Next, we examined EMT factors downstream of TNF-α. Among three EMT transcription factors (Snail, Twist1, and Zeb2), the mRNA level of *SNAI1* was decreased by ACY-241 and JQ1 (Figure S1H–J). Moreover, protein expression of Snail was moderately reduced by inhibition of BET and HDAC6 (Figure 4A,B). TNF-α-induced EMT in HNSCC is known to be involved in the nuclear factor-κB (NF-κB) signaling pathway, and NF-κB activation by TNF-α requires AKT phosphorylation [31]. Thus, we investigated whether ACY-241 and JQ1 affect the AKT/NF-κB signaling pathway in HPV-positive and HPV-negative HNSCC cells. HDAC6 and BET co-inhibition synergistically blocked phosphorylation of AKT and p65, which positively correlates with HNSCC malignancy [32], without changing total ATK and p65 levels (Figure 4C,D). Together, our results indicate that the combination of ACY-241 and JQ1 suppresses TNF-α-induced responses by dysregulating MT1-MMP, MMP-2, and MMP-9 and by modulating AKT/NF-κB signaling pathways in both HPV-positive and HPV-negative HNSCC cells.

### 2.4. Combination Treatment of ACY-241 and JQ1 Synergistically Impairs Migration and Invasion in HNSCC Cells

**Figure 5:**
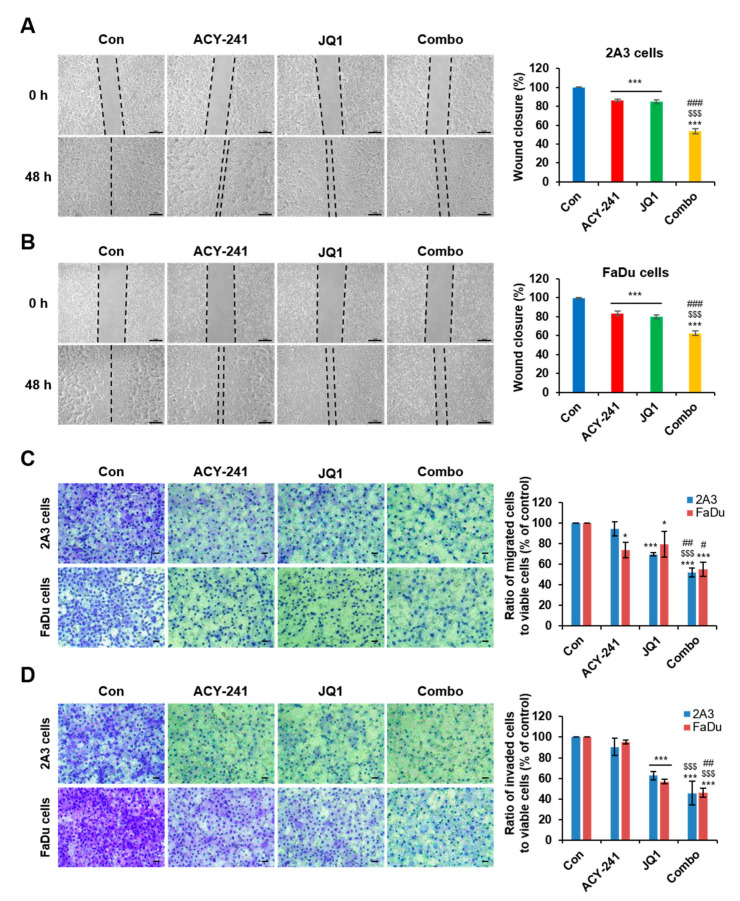
Combination treatment of ACY-241 and JQ1 synergistically inhibit HNSCC cell invasion and migration. (**A**,**B**) Wound healing assay of 2A3 and FaDu cells. Cells were seeded in 6-well plates and then scratched after cell adhesion. Artificial lines were drawn on microscopic images to visualize the scratched area. Percentage of wound closure was determined by the difference between wound areas in 0 h and 48 h. The wound was photographed at 50× magnification. Scale bar = 50 μm. (**C**) Transwell migration assay and (**D**) transwell invasion assay of 2A3 and FaDu cells. The cells were photographed at 200× magnification. Scale bar = 100 μm. Matrigel (0.4 mg/mL) was coated on the inner inserts of transwell plate for 1 h during invasion assay. Cells were treated with 0.2% DMSO, ACY-241 (4 μM), and JQ1 (2 μM) alone or in combination for 48 h. Data were normalized by cell viability to represent migration and invasion of viable cells. Values represent mean ± SD (*n* = 3). * *p* < 0.05, or *** *p* < 0.001 vs. DMSO control, ^$$$^ *p* < 0.001 vs. ACY-241-treated group, ^#^ *p* < 0.05, ^##^ *p* < 0.01 or ^###^ *p* < 0.001 vs. JQ1-treated group.

Next, we performed wound healing assay and transwell migration assay to examine the migration of tumor cells. After 48 h of combination treatment in 2A3 and FaDu cells, proliferation and wound closure were synergistically diminished, and a significantly small number of cells migrated across the transwell membrane (Figure 5A–C). Then, we performed transwell invasion assay by coating the insert membrane with Matrigel and providing ECM-like circumstances to study cell invasion. Similar to the results of transwell migration assay, combination treatment decreased the penetration of cells through the membrane (Figure 5D). To represent migration and invasion capacity of viable cells, the absorbance of cells has been normalized by cell viability from Figure 1. These results suggest that co-targeting HDAC6 and BET weakens the migration and invasion capacity of HPV-positive and HPV-negative HNSCC cells. 

## 3. Discussion

**Figure 6:**
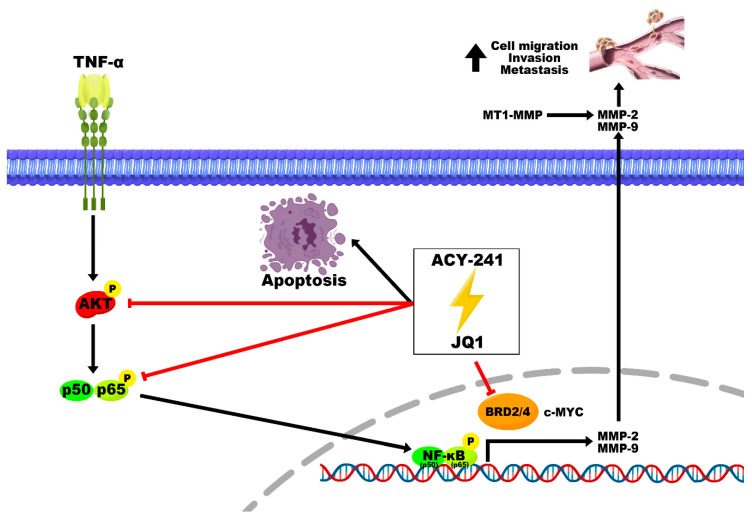
Schematic diagram of post-translational modulations underlying synergistic anticancer effects induced by ACY-241 and JQ1 combination treatment in HNSCC. ACY-241 and JQ1 dysregulate MMP-2 and MMP-9 expression and secretion by suppressing TNF-α, an inducer of active AKT and NF-κB. Migration, invasion, and metastasis of HNSCC cells are synergistically inhibited by ACY-241 and JQ1 treatments.

Combination therapy is an economic therapeutic strategy that combines two or more treatment agents that have already been developed. This approach exhibits more significant therapeutic effects by regulating tumor growth, metastasis, cell cycle, and apoptosis while escaping drug resistance evoked by monotherapies [33]. Synergistic effects of HDAC and BET inhibitors have been observed in multiple cancers [34,35]. In multiple myeloma, sensitivity to JQ1 is increased after cotreatment with HDAC6 inhibitor as confirmed through cell proliferation inhibition and apoptosis promotion [36]. However, the combinational treatment of HDAC6 and BET inhibitors has not yet been studied in HNSCC. The Kaplan–Meier plot showing the overall survival (OS) of HNSCC patients regarding gene expressions of HDAC6, BRD2, and BRD4 demonstrates that patients with high BRD2 or BRD4 display low survival (Figure S3). In addition, previous studies have demonstrated that HPV-positive HNSCC patients have higher OS than HPV-negative HNSCC patients [37]. Here, we demonstrate that the combination of HDAC6-specific inhibitor ACY-241 and BET inhibitor JQ1 modulates molecular mechanisms of apoptosis and metastasis, displaying more significant synergistic anticancer effects than the additive effect of single inhibitors (Figure 6). We showed that this combination approach reduces cell growth and viability and activates the caspase-3-dependent apoptosis signaling pathway. Anti-apoptotic protein Bcl-xL is downregulated to promote apoptosis, but other Bcl-2 associated pro-apoptotic proteins remain unchanged by ACY-241 and JQ1 combination (Figure S2). Moreover, we show by flow cytometry that co-inhibition of HDAC6 and BET causes apoptosis in HNSCC cells. 

Previous studies have identified the critical role of MMP-2 and MMP-9 in tumor invasion and lymph node metastasis of HNSCC [10]. In hypopharyngeal cancer tissues, mRNA and protein levels of MMP-9 and MMP-2 are significantly upregulated compared to paracancerous tissues. Moreover, the expression levels are enhanced with the increased lymph node metastasis degree and tumor clinical stages, suggesting that MMP-2 and MMP-9 are involved in the occurrence and aggressiveness of hypopharyngeal cancer [38]. It has been suggested that MMP-2 and MMP-9 are potential prognostic biomarkers of HNSCC [39]. We show that combination therapy of ACY-241 and JQ1 has significant anti-metastatic effects through MMP-2 and MMP-9 modulations. This pathway features the suppression of TNF-α/AKT/NF-κB signaling cascade. TNF-α is a multifunctional cytokine that promotes EMT by activating AKT and NF-κB, resulting in augmented invasion and metastasis in many cancers, including OSCC cells [40]. Correspondingly, metastatic tongue squamous cell carcinoma tissues and cells are detected with highly active NF-κB [41]. NF-κB directly binds to 5’-flanking region of the MT1-MMP gene and MMP-9 promoter, thereby inducing MMP-2 and MMP-9 expressions for dynamic EMT [42,43]. Moreover, active PI3K/AKT increases MMP-9-mediated EMT in laryngeal cancer cells [44]. In consistent with previous findings, we demonstrate that the combination of small molecule inhibitors synergistically suppresses metastasis by modulating MMP-2 and MMP-9 expressions via TNF-α/AKT/NF-κB signaling in HPV-negative and HPV-positive HNSCC.

Blocking of the interaction of HSP90 and MMP-2 and MMP-9 induces metastasis suppression in breast cancer [45] and lung cancer [46], respectively. The dissociation of MMP and HSP90 is caused by the hyperacetylation of HSP90, which is a substrate of HDAC6 [46]. Thus, suppression of migration and invasion is triggered by promoting HDAC6-mediated HSP90/MMP-2 or MMP-9 dissociation and followed by MMP-2 and MMP-9 degradation in breast and lung cancer cells. Consistent with the previous findings, HDAC6 inhibition by ACY-241 blocks migration and invasion of HNSCC cells. In the case of HNSCC, ACY-241 significantly inhibits MMP-2 expression more than it does MMP-9. It has been previously reported that the expression level of MMP-2 is higher than that of MMP-9 in FaDu cells [47]. Moreover, MMP-2 is more correlated with lymph node metastasis than MMP-9 in laryngeal squamous cell carcinoma [48]. These data imply that targeting MMP-2 may be more effective for metastasis suppression than targeting MMP-9 in HNSCC. In addition to ACY-241, although the mechanism is unclear, JQ1 downregulates the expression of MMP2- and MMP-9 and inhibits angiogenesis in glioblastoma tumors [49] and pancreatic ductal adenocarcinoma cells [50]. Here, we demonstrate for the first time that combined treatment of ACY-241 and JQ1 synergistically blocks migration and invasion of HPV-positive and HPV-negative HNSCC cells by degrading MMP2 and MMP-9. These findings suggest that a combination of BET and HDAC6 inhibitors significantly suppresses metastasis in HNSCC irrespective of HPV infection. 

Further investigations are required to confirm which member of BET is deregulated by JQ1 to induce anticancer effects. Many studies of JQ1 have focused on BRD4 inhibition because BRD4 is an appealing target in cancer treatment. JQ1 is often called a BRD4 inhibitor due to its strong inhibitory potency for BRD4. However, JQ1 is a pan-BET inhibitor that can moderately inhibit BRD2 by binding to the acetyl-lysine binding pocket of bromodomains [51]. According to our Gene Expression Omnibus (GEO) analysis, BRD2 was significantly overexpressed and positively correlated with HDAC6 in HNSCC patients among four BET proteins (Figure S4). This suggests that BRD2 inhibition may synergize with HDAC6 inhibition to suppress metastasis in HNSCC. This hypothesis is supported by the study that ZNF281, an EMT transcription factor, is enriched in the BRD2-binding genes that are downregulated by JQ1 [52]. It is also known that BRD2 promotes EMT, while BRD3 and BRD4 negatively regulate this process in triple-negative breast cancer. Thus, BRD2 knockdown or JQ1 treatment reduced the transcription of genes involved in EMT [53]. For these reasons, it is necessary to examine which BET protein binds to the EMT-promoting genes observed in this study (MMPs, TNF-α, AKT, NF-κB) and whether BRD2 overexpression participates in HNSCC metastasis. 

In cell growth and viability assays, we obtained greater GI_50_ and IC_50_ values for HPV-positive HNSCC cells (Table 1). However, this result was not enough to determine that HPV-positive HNSCC cells have higher sensitivity for ACY-241 and JQ1, because a similar degree of synergistic anticancer effects was observed in subsequent experiments. We have shown by GEO analysis that the *BRD2* mRNA level is upregulated and displays a stronger positive correlation with *HDAC6* mRNA in HPV-positive HNSCC samples compared to HPV-negative samples (Figure S4). In addition, we interestingly observed that adhesion and migration abilities in HPV-positive 2A3 cells are more impaired compared with those in HPV-negative FaDu cells in the cell culture. This observation can be explained by HPV E6 oncogene expression because E6 disrupts the cell adhesion pathway by TAp63β degradation [54]. Although the significant difference derived from HPV infection is not clearly demonstrated, further investigations are required to uncover the role of HPV infection in metastasis and survival outcome of HNSCC. 

## 4. Materials and Methods 

### 4.1. Reagents

ACY-241 (Citarinostat) and JQ1 were purchased from Selleck Chemicals (Houston, TX, USA). Powders were solubilized in DMSO (Sigma Chemical, St. Louis, MO, USA) to make 50 mM stocks. Antibodies against α-tubulin (sc-32293), c-Myc (sc-40), Snail (sc-28199), p-AKT (sc-7985-R), AKT (sc-8312), p65 (sc-8008), and TNF-α (sc-52746) were purchased from Santa Cruz Biotechnology (Santa Cruz, CA, USA). Antibodies against GAPDH (AP0066) and p-p65 (BS4138) were from Bioworld Technology (Bloomington, MN, USA). Antibodies against MMP-2 (A2454) and MMP-9 (A0289) were from ABclonal Technology (Woburn, MA, USA). Antibodies against PARP (551024) and XIAP (610716) were from BD Biosciences (San Jose, CA, USA). Antibodies against caspase-3 (#9662), Bcl-xL (#2762), and MT1-MMP (#13130) were from Cell Signaling Technology (Danvers, MA, USA). Antibody against acetyl α-tubulin (T6793) was from Sigma-Aldrich (St. Louis, MO, USA). Antibody against HDAC6 was from Bethyl Laboratories (Montgomery, TX, USA). 

### 4.2. HNSCC Cell Lines and Culture

The hypopharyngeal cancer cell lines were used to represent HNSCC cell lines. HPV-positive 2A3 cells and HPV-negative FaDu cells were bought from American Type Culture Collection (ATCC). 2A3 and FaDu cells were cultured in Dulbecco’s Modified Eagle Medium (Sigma Chemical, St. Louis, MO, USA) supplemented with 10% fetal bovine serum (FBS), 100 U/mL penicillin, and 100 μg/mL streptomycin. Cells were subcultured every 3–4 days and incubated in a humidified atmosphere at 37 °C and 5% CO_2_. 

### 4.3. Cell Growth and Viability Assay

Cell growth and viability were determined using CCK-8 assay. Each cell line was seeded at a density of 3 × 10^3^ cells in 96-well plates containing 130 μL medium. After overnight incubation, cells were treated with ACY-241 and JQ1 in single and in combination, then incubated for another 24, 48, and 72 h. Thirteen microliters of WST-8 was added to each well, and absorbance was measured at 450 nm using a multimode microplate reader (Tecan Group, Ltd., Mannedorf, Switzerland).

### 4.4. Drug Combination Analysis

Synergism between ACY-241 and JQ1 was evaluated using Chou-Talalay method [24]. Fraction-affected (Fa) versus combination index (CI) plot was drawn using CalcuSyn (Biofosft). The drug combination was considered synergistic when CI was less than 1.

### 4.5. Apoptosis Assay

Flow cytometry analysis (FACS) was used to analyze apoptosis. Each cell line was seeded at a density of 7 × 10^5^–1 × 10^6^ cells in 100 mm cell culture dish and treated with 4 μM ACY-241 and 2 μM JQ1 on the next day. After 48 h incubation, cells were washed twice with ice-cold phosphate-buffered saline (PBS) and detached from the plate with trypsin and EDTA. Cell pellets were collected after 3 min centrifugation at 1000 rpm, then resuspended with 4–700 μL of 1× binding buffer. Cells were stained with 5 μL propidium iodide (PI) and 0.5 μL of Annexin V-fluorescein isothiocyanate (FITC) in the dark for 15 min and diluted with 400 μL of 1× binding buffer (Annexin V-FITC Apoptosis Detection Kit; BD556547, BD Pharmingen San Diego, CA, USA). The cells were analyzed using a fluorescence-activated cell sorting flow cytometer.

### 4.6. Wound Healing Assay

2A3 and FaDu cells were seeded in a 6-well plate at a density of 7 × 10^5^~1.5 × 10^6^ cells. On the next day, cells were scratched with 200 μL pipette tip and washed twice with serum-free medium, followed by treatment of 4 μM ACY-241 and 2 μM JQ1. The wound was photographed after 0 h and 48 h incubation at 50× magnification. 

### 4.7. Transwell Migration and Invasion Assay

Transwell migration and invasion assays were performed using a transwell insert plate (PET membrane, 8 μM pore, SPL 36224, Korea). Transwell invasion assay was conducted after coating the insert membrane with 0.4 mg/mL Matrigel (Corning Matrigel Membrane Matrix, cat# 356234), and transwell migration assay was conducted without coating. Four hundred microliters of cell suspension solution in serum-free medium was added to the upper well at a density of 2 × 10^5^ cells, and 500 μL of medium containing 10% FBS was added to the lower well. On the next day, 4 μM ACY-241 and 2 μM JQ1 were added to the lower well. After 48 h incubation, interiors of the inserts were cleaned by wet cotton swabs. Cells were stained with the cell stain solution (0.1% crystal violet, 20% methanol) and visualized under a light microscope at 200× with iSolution Lite (IMT i-Solution Inc., BC, Canada). Then, cells were solubilized in 10% glacial acetic acid, and absorbance was measured at 560 nm using a multimode microplate reader (Tecan Group, Ltd., Mannedorf, Switzerland). To represent migration and invasion capacity of viable cells, the absorbance of cells has been normalized by cell viability.

### 4.8. Western Blot Analysis

2A3 and FaDu cells were seeded at a density of 5 × 10^5^ cells and treated with 4 μM ACY-241 and 2 μM JQ1 on the next day. Cells were washed twice with ice-cold PBS and extracted with 100 μL lysis buffer, then lysed by sonication at 20% amplitude. Bradford protein assay was performed to measure protein concentrations. Protein samples were prepared with a 5× sample buffer and loaded to 7.5–12% polyacrylamide gel. After SDS-page, proteins were transferred to nitrocellulose membrane. Membranes were blocked with 5% skim milk at room temperature and incubated with primary antibody against α-tubulin (1:1000), HDAC6 (1:1000), c-Myc (1:250), p-AKT (1:500), AKT (1:1000), p65 (1:1000), TNF-α (1:250), GAPDH (1:10,000) and p-p65 (1:1000), MMP-2 (1:500), MMP-9 (1:1000), PARP (1:1000), XIAP (1:1000), caspase-3 (1:500), Bcl-xL (1:500), MT1-MMP (1:500), acetyl α-tubulin (1:2000), and Snail (1:2000) overnight at 4 °C. Membranes were washed with 0.1% Tween-20/PBS and incubated for 3 h with an anti-rabbit/mouse secondary antibody coupled to HRP. Bound antibodies were detected with the ECL Western blotting analysis system (Thermo Scientific Pierce). 

### 4.9. Statistical Analysis

All results are expressed as means ± standard deviation (SD) of three independent experiments. For the cell growth and viability test of single agents, statistical significance was determined by unpaired two-tailed Student’s *t*-test. Statistical analysis for other data was performed by GraphPad Prism software 7.0 (Graphpad Software, San Diego, CA, USA). One-way or two-way ANOVA followed by post-hoc analysis with Bonferroni’s multiple comparison test was used to evaluate statistical significance. *p* < 0.05 was considered statistically significant for data.

## 5. Conclusions

Collectively, we demonstrate that the combined targeting of HDAC6 and BET in HNSCC synergistically promotes apoptosis and suppresses cell growth and viability, migration, and invasion. ACY-241 and JQ1 induce apoptosis by modulating anti-apoptotic proteins. Importantly, this combination approach regulates protein levels of MMP-2 and MMP-9 via TNF-α/AKT/NF-κB axis to impair metastasis of HPV-positive HNSCC and HPV-negative HSNCC cells. Simultaneously inhibiting HDAC6 and BET, which potentially drive tumors to advanced stages, could be a promising strategy to treat head and neck cancer.

## Figures and Tables

**Table 1:** IC_50_ and GI_50_ values of ACY-241 and JQ1 in 2A3 and FaDu cells.

Time	72 h			
Drug	ACY-241		JQ1	
Cell Line	2A3	FaDu	2A3	FaDu
^1^ IC_50_ (μM)	3.978	9.190	4.163	9.640
^2^ GI_50_ (μM)	3.986	9.191	4.163	9.645

^1^ IC_50_: half maximal inhibitory concentration; ^2^ GI_50_: half maximal growth inhibition concentration.

## Supplementary Materials

Supplementary materials can be found at https://www.mdpi.com/1422-0067/21/18/6873/s1. Supplementary Figure S1: Relative mRNA expressions analyzed by qRT-PCR in 2A3 and FaDu cells. (A–C) Target genes of ACY-241 and JQ1. (D–J) Genes of MMP family and EMT-TFs. Total RNA was extracted after 24 h of ACY-241 (4 μM) or JQ1 (2 μM) treatment alone or in combination. qPCR data are normalized by GAPDH gene expression. Detailed methods and primer sequences used for qPCR are attached in Supporting Information. Values represent mean ± SD (*n* = 2). * *p* < 0.05, ** *p* < 0.01, or *** *p* < 0.001 vs. DMSO control, ^$^ *p* < 0.05, ^$$^ *p* < 0.01, or ^$$$^ *p* < 0.001 vs. ACY-241-treated group, ^##^ *p* < 0.01 vs. JQ1-treated group. ns = not significant. Supplementary Figure S2: Bcl-2 related pro-apoptotic proteins are unaffected by ACY-241 and JQ1 treatments. Immunoblot analysis of Bak, Bax, and Bad in (A) 2A3 cells and (B) FaDu cells. Total protein was extracted after 24 h of ACY-241 (4 μM) or JQ1 (2 μM) treatment alone or in combination. α-tubulin was used as a loading control. Protein levels were quantified relative to the loading control. Primary antibodies against Bak (sc-832) and Bad (sc-8044) were purchased from Santa Cruz Biotechnology (Santa Cruz, CA), and Bax (#2772) was from Cell Signaling Technology (Danvers, MA). Indicated antibodies were diluted in 1:1000 ratio with 5% skim milk. Figure S3: Kaplan-Meier plots for overall survival (OS) of HNSCC patients. (A) Kaplan–Meier plot regarding gene expression of HDAC6. (B) Kaplan–Meier plot regarding gene expression of BRD2. (C) Kaplan–Meier plot regarding gene expression of BRD4. Low and high percentile = 50. Data were obtained from OncoLnc dataset (http://www.oncolnc.org/). Supplementary Figure S4: Gene expression analysis of HDAC6, BRD2, and BRD4. (A–C) Relative gene expression level of HDAC6, BRD2, and BRD4 in normal and HNSCC patient samples. ** *p* < 0.01 vs. normal samples. (D–F) Relative gene expression level of HDAC6, BRD2, and BRD4 in HPV-positive and HPV-negative HNSCC samples. * *p* < 0.05, ** *p* < 0.01 or *** *p* < 0.001 vs. HPV-negative HNSCC samples. ns = not significant. (G) BRD2 mRNA expression and its association with HDAC6 mRNA in HNSCC patient samples based on Pearson’s correlation. (H) BRD2 mRNA expression and its association with HDAC6 in HPV-positive HNSCC samples based on Pearson’s correlation. (I) BRD2 mRNA expression and its association with HDAC6 in HPV-negative HNSCC samples based on Pearson’s correlation. Genomic array data of BRD2 and HDAC6 were obtained from NCBI GEO under accession number (A–C, G) GDS2520 (n = 22 for normal, n = 22 for HNSCC) and (D–F, H–I) GDS1667 (n = 8 for HPV-positive HNSCC, n = 28 for HPV-negative HNSCC). Gene expression profiles were compared using Mann–Whitney and Pearson’s correlation tests. Values are mean ± SD from independent samples.

## Abbreviations

BET	Bromodomain and extraterminal
BRD	Bromodomain
CI	Combination index
EMT	Epithelial–mesenchymal transition
HNSCC	Head and neck squamous cell carcinoma
HPV	Human papilloma virus
HDAC6	Histone deacetylase 6
MMP	Matrix metalloproteinase
MT1-MMP	Membrane-type 1-matrix metalloproteinase
NF-κB	Nuclear factor-κB
OPSCC	Oropharyngeal squamous cell carcinoma
TNF-α	Tumor necrosis factor-α
