# Supplementary material for: Combination of ACY-241 and JQ1 Synergistically Suppresses Metastasis of HNSCC via Regulation of MMP-2 and MMP-9
Source: Int J Mol Sci. 2020 Sep 19;21(18):6873. doi: 10.3390/ijms21186873 (PMC7554925; doi:10.3390/ijms21186873)
Supplement: Supplementary file 1 [file ijms-21-06873-s001.pdf]

Cho et al. Figure S1

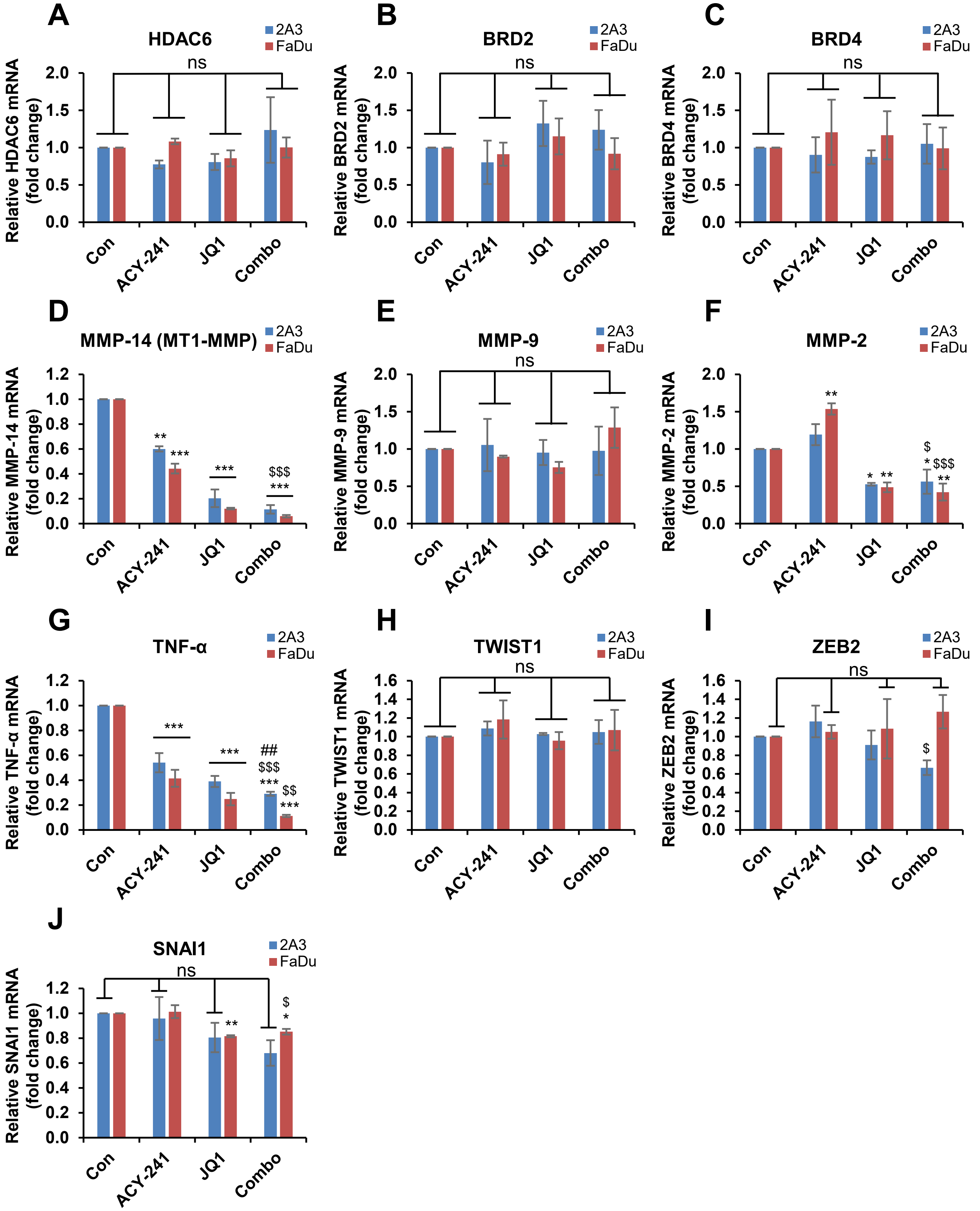

Figure S1. Relative mRNA expressions analyzed by qRT-PCR in 2A3 and FaDu cells. **(A-C)** Target genes of ACY-241 and JQ1. **(D-J)** Genes of MMP family and EMT-TFs. Total RNA was extracted after 24 h of ACY-241 (4 μM) or JQ1 (2 μM) treatment alone or in combination. qPCR data are normalized by GAPDH. Detailed methods and primer sequences used for qPCR are attached in Supporting Information. Values represent mean ± SD (n = 2). * $p < 0.05$, ** $p < 0.01$, or *** $p < 0.001$ vs. DMSO control, $^{\$}$ $p < 0.05$, $^{\$\$}$ $p < 0.01$, or $^{\$\$\$}$ $p < 0.001$ vs. ACY-241-treated group, $^{\#\#}$ $p < 0.01$ vs. JQ1-treated group. ns = not significant.

Cho et al. Figure S2

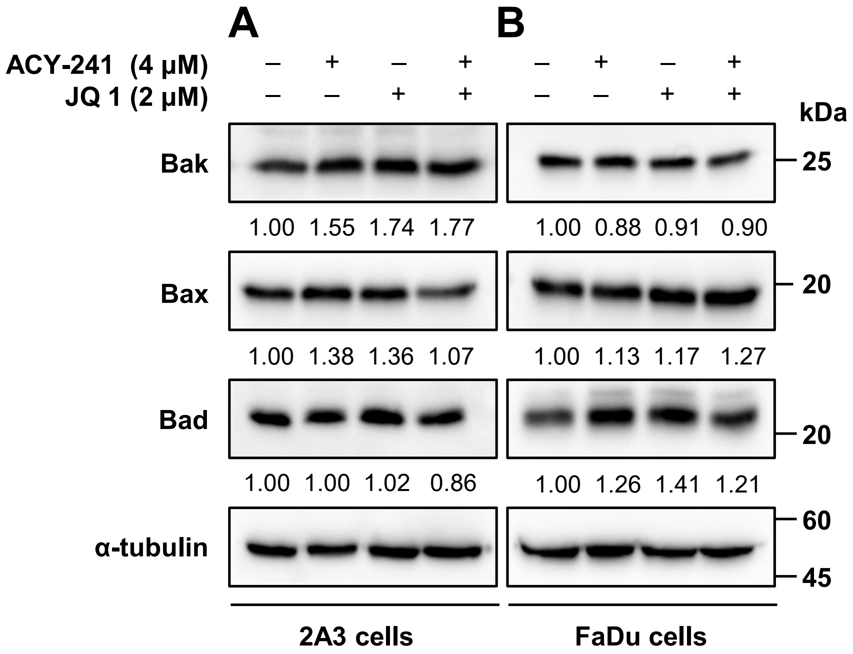

Figure S2. Bcl-2 related pro-apoptotic proteins are unaffected by ACY-241 and JQ1 treatments. Immunoblot analysis of Bak, Bax, and Bad in **(A)** 2A3 cells and **(B)** FaDu cells. Total protein was extracted after 24 h of ACY-241 (4 μM) or JQ1 (2 μM) treatment alone or in combination. α-tubulin was used as a loading control. Protein levels were quantified relative to the loading control. Primary antibodies against Bak (sc-832) and Bad (sc-8044) were purchased from Santa Cruz Biotechnology (Santa Cruz, CA) and Bax (#2772) was from Cell Signaling Technology (Danvers, MA). Indicated antibodies were diluted in 1:1,000 ratio with 5% skim milk.

Cho et al. Figure S3

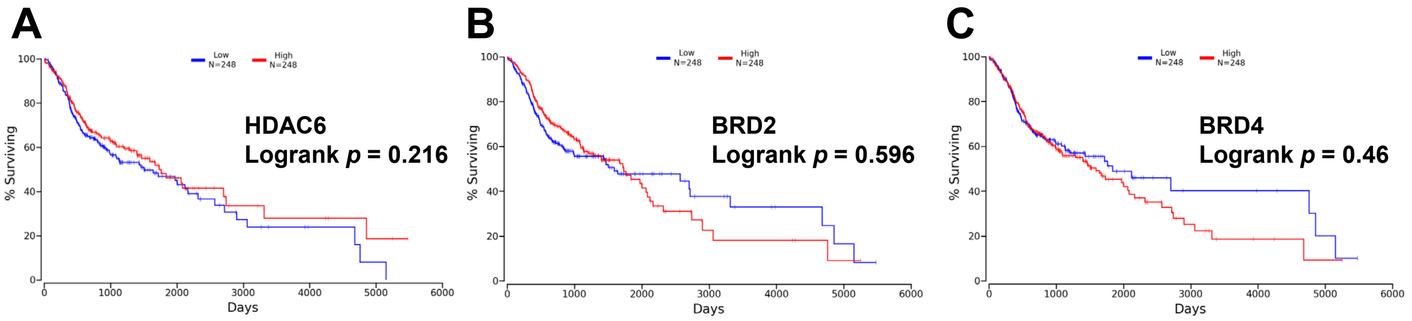

Figure S3. Kaplan-Meier plots for overall survival (OS) of HNSCC patients. **(A)** Kaplan-Meier plot regarding gene expression of HDAC6. **(B)** Kaplan-Meier plot regarding gene expression of BRD2 **(C)** Kaplan-Meier plot regarding gene expression of BRD4. Low & high percentile = 50. Data was obtained from oncolnc.org.

Cho et al. Figure S4

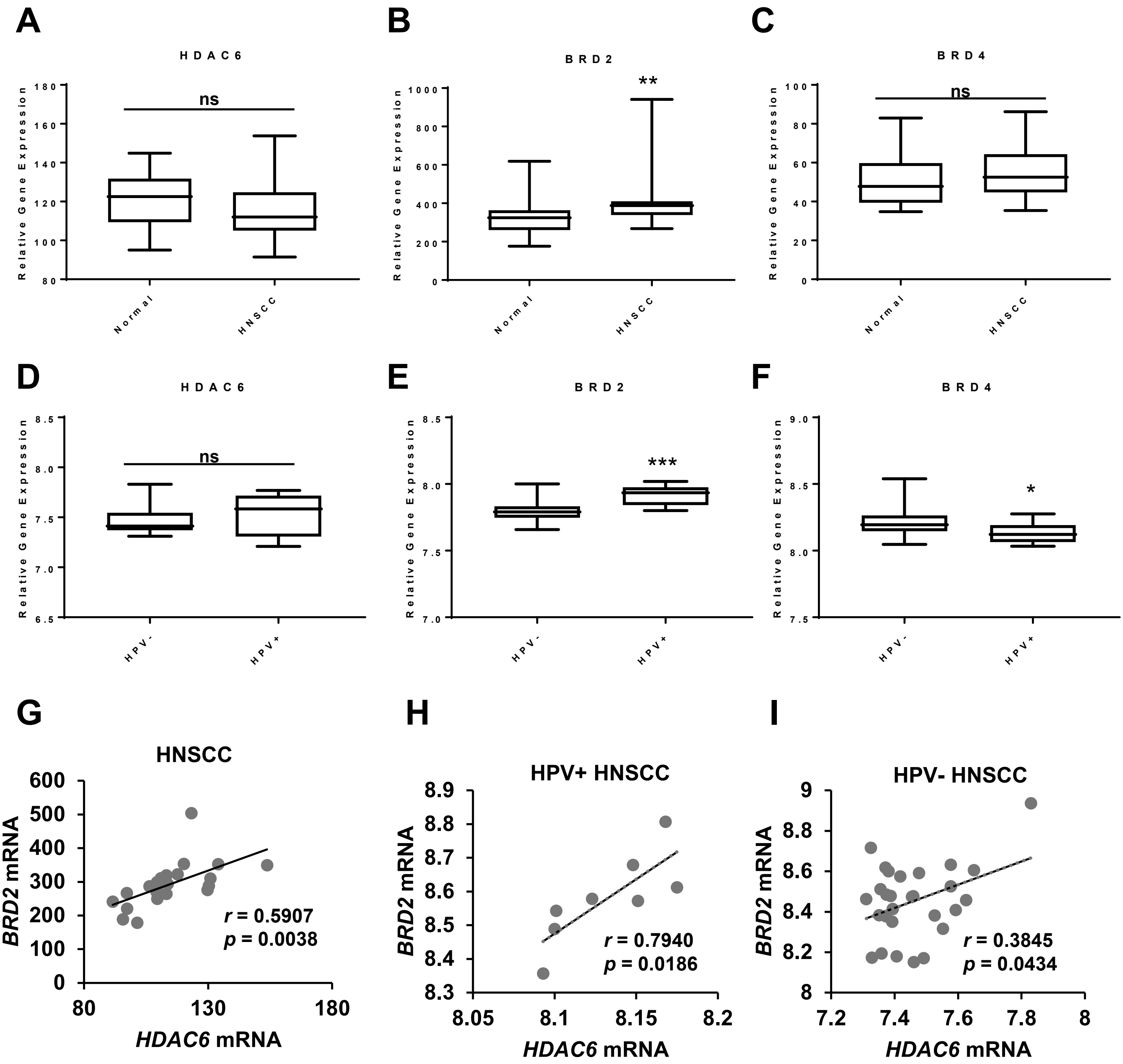

Figure S4. Gene expression analysis of HDAC6, BRD2, and BRD4. **(A-C)** Relative gene expression level of HDAC6, BRD2, and BRD4 in normal and HNSCC patient samples. ** $p < 0.01$ vs. normal samples. **(D-F)** Relative gene expression level of HDAC6, BRD2, and BRD4 in HPV-positive and HPV-negative HNSCC samples. * $p < 0.05$ or *** $p < 0.001$ vs. HPV-negative HNSCC samples. ns = not significant. **(G)** BRD2 mRNA expression and its association with HDAC6 mRNA in HNSCC patient samples based on Pearson's correlation. **(H)** BRD2 mRNA expression and its association with HDAC6 in HPV-positive HNSCC samples based on Pearson's correlation. **(I)** BRD2 mRNA expression and its association with HDAC6 in HPV-negative HNSCC samples based on Pearson's correlation. Genomic array data of BRD2 and HDAC6 were obtained from NCBI GEO under accession number **(A-C, G)** GDS2520 (n = 22 for normal, n = 22 for HNSCC) and **(D-F, H-I)** GDS1667 (n = 8 for HPV-positive HNSCC, n = 28 for HPV-negative HNSCC). Gene expression profiles were compared using Mann-Whitney and Pearson's correlation tests. Values are mean ± SD from independent samples.
